# A Three-time Winner

**Published:** 1887-12

**Authors:** 


### "A THREE-TIME WINNER."

### Has Hanlan Lost His Grip?—Philosophical Training Demanded.

The defeat of "Ned" Hanlan by Teemer at Toronto in August indicates the "end of the glory" of the doughty champion.

He has sustained his record with admirable pluck and success, but the tremendous strain of years of training must certainly some day find its limit.

Apropos of this we recall the following interesting reminiscence of aquatic annals.

On a fine, bright day in August, 1871, an excited multitude of 15,000 to 20,000 persons lined the shores of the beautiful Kenebecassis, near St. John, N. B., attracted by a four-oared race between the famous Paris crew of that city and a picked English crew for $5,000 and the championship of the world. Wallace Ross, the present renowned oarsman, pulled stroke for the Blue Nose crew, and "Jim" Renforth, champion sculler and swimmer of England and of the world, was stroke in the English shell.

Excitement was at fever heat.

But three hundred yards of the course had been covered when the Englishmen noticed that their rivals were creeping away.

"Give us a dozen, Jim," said the veteran Harry Kelly, ex-champion of England, who was pulling No. 3. oar.

"I can't, boys, I'm done," said Renforth, and with these words he fell forward, an inanimate heap in the boat.

"He has been poisoned by book-makers," was the cry, and belief.

Everything that science and skill could suggest for his restoration was tried; but after terrible struggles of agony, the strong man, the flower of the athletes and pride of his countrymen, passed away.

The stomach was analyzed but no sign or trace of poison could be found therein, though general examination showed a very strange condition of the blood and the life giving and health preserving organs caused by years of unwise training. While the muscular development was perfect the heart and kidneys were badly congested.

The whole system was, therefore, in just that state when the most simple departure from ordinary living and exertion was of momentous consequence. His wonderful strength only made his dying paroxysms more dreadful and the fatality more certain.

Hanlan is now in Australia. Beach, champion of that country, is a powerful fellow, who probably understands the liability of athletes to death from over-training, the effect thereof being very serious on the heart, blood and kidneys, as shown by poor Renforth's sudden death.

Within the past three years he has taken particular care of himself, and when training, always reinforces the kidneys and prevents blood congestion in them and the consequent ill effect on the heart by using Warner's safe cure, the sportsman's universal favorite, and says he "is astonished at the great benefit."

Harry Wyatt, the celebrated English trainer of athletes, who continues himself to be one of the finest specimens of manhood and one of the most successful of trainers, writes over his own signature to the English *Sporting Life*, September 5th, saying: "I consider Warner's safe cure invaluable for all training purposes and outdoor exercise. I have been in the habit of using it for a long time. I am satisfied that it pulled me through when nothing else would, and it is always a three-time winner!"

Beach's and Wyatt's method of training is sound and should be followed be all.

